# Supplementary material for: Analysis of related factors for adolescents’ intention to use alcohol in Korea
Source: PLoS One. 2022 Oct 11;17(10):e0275957. doi: 10.1371/journal.pone.0275957 (PMC9553045; doi:10.1371/journal.pone.0275957)
Supplement: Attachment — Submitted filename: PONE-D-21-30940 (1).pdf [file pone.0275957.s001.pdf]

PLOS ONE

Analysis of related factors for adolescents’ intention to use alcohol in Korea

--Manuscript Draft--

**Manuscript Number:**	PONE-D-21-30940
**Article Type:**	Research Article
**Full Title:**	Analysis of related factors for adolescents’ intention to use alcohol in Korea
**Short Title:**	Analysis of related factors for adolescents’ intention to use alcohol in Korea
**Corresponding Author:**	Aeri Jung<br>Bucheon University<br>Bucheon, KOREA, REPUBLIC OF
**Keywords:**	Alcohol, Public health, Intention to use alcohol, Drinker, Non-drinker, Adolescent, Environment, Policy
**Abstract:**	<p class="MDPI17abstract" style="margin:0cm;margin-bottom:.0001pt;text-indent: 40.0pt;line-height:150%">The harmful effects of alcohol consumption by adolescents are increasingly emphasized. It is thus necessary to identify individual and environmental factors that encourage such drinking. This study investigated factors associated with the sustainable use of alcohol (SUA) in adolescents who consume alcohol, and the possibility of future drinking (PFD) in non-drinking adolescents. Data from 'the Adolescents Awareness Survey of Alcohol Encouraging Environment' by the Ministry of Health and Welfare and the Korea Health Promotion Institute (2017) were used. The survey was completed by 1038 subjects, selected through a proportional allocation extraction method, who were aged 13-18 years and lived in five cities with a population of over 1 million. The factors associated with SUA included gender (β=0.634, p<0.004), grade, drinking alone (β=-2.147, p<0.002), liquor advertising (β=1.644, p<0.0001), ease of alcohol purchase (β=1.541, p<0.025), parental permission to drink (β=1.084, p<0.0001), not knowing of mother’s education level (β=-0.685, p<0.045), positive expectancies from drinking (β=0.141, p<0.0001), and the number of pubs (β=0.303, p<0.002), internet game cafes (β=0.456, p<0.019) and Karaokes (β=-0.098, p<0.023) in the community. The factors associated with the possibility of future drinking (PFD) in non-drinkers were grade, liquor advertising (β=-1.355, p<0.001), parental permission to drink (β=0.783, p<0.020), positive expectancies from drinking alcohol (β=0.139, p<0.0001) and the relationship with father (β=0.072, p<0.033). Multidimensional interventions, including those by individuals, parents, peers, and local communities, are needed therefore to prevent sustainable use of alcohol (SUA) and the possibility of future drinking (PFD) in adolescents.<o:p></o:p></p>
**Order of Authors:**	Eun-A Park
	Aeri Jung
	Sungyong Choi
**Additional Information:**	
**Question**	**Response**
**Financial Disclosure**<br><br>Enter a financial disclosure statement that describes the sources of funding for the work included in this submission. Review the submission guidelines for detailed requirements. View published research articles from *PLOS ONE* for specific examples.	The author(s) received no specific funding for this work.

Powered by Editorial Manager® and ProduXion Manager® from Aries Systems Corporation

This statement is required for submission and **will appear in the published article** if the submission is accepted. Please make sure it is accurate.<br><br>**Unfunded studies**<br>Enter: *The author(s) received no specific funding for this work.*<br><br>**Funded studies**<br>Enter a statement with the following details:<br>• Initials of the authors who received each award<br>• Grant numbers awarded to each author<br>• The full name of each funder<br>• URL of each funder website<br>• Did the sponsors or funders play any role in the study design, data collection and analysis, decision to publish, or preparation of the manuscript?<br>• **NO** - Include this sentence at the end of your statement: *The funders had no role in study design, data collection and analysis, decision to publish, or preparation of the manuscript.*<br>• **YES** - Specify the role(s) played.<br><br>* typeset	
**Competing Interests**<br><br>Use the instructions below to enter a competing interest statement for this submission. On behalf of all authors, disclose any competing interests that could be perceived to bias this work—acknowledging all financial support and any other relevant financial or non-financial competing interests.<br><br>This statement is required for submission and **will appear in the published article** if the submission is accepted. Please make sure it is accurate and that any funding sources listed in your Funding Information later in the submission form are also declared in your Financial Disclosure statement.<br><br>View published research articles from	The authors have declared that no competing interests exist.

PLOS ONE for specific examples.<br><br>**NO authors have competing interests**<br><br>Enter: *The authors have declared that no competing interests exist.*<br><br>**Authors with competing interests**<br><br>Enter competing interest details beginning with this statement:<br><br>*I have read the journal's policy and the authors of this manuscript have the following competing interests: [insert competing interests here]*<br><br>* typeset	
**Ethics Statement**<br><br>Enter an ethics statement for this submission. This statement is required if the study involved:<br><br>• Human participants<br>• Human specimens or tissue<br>• Vertebrate animals or cephalopods<br>• Vertebrate embryos or tissues<br>• Field research<br><br>Write "N/A" if the submission does not require an ethics statement.<br><br>General guidance is provided below. Consult the submission guidelines for detailed instructions. **Make sure that all information entered here is included in the Methods section of the manuscript.**	This study were reviewed by the Bioethics Committee of the Korea Institute for Health Promotion and Development (IRB 1709-HR-010-01).

**Format for specific study types**<br><br>**Human Subject Research (involving human participants and/or tissue)**<br>• Give the name of the institutional review board or ethics committee that approved the study<br>• Include the approval number and/or a statement indicating approval of this research<br>• Indicate the form of consent obtained (written/oral) or the reason that consent was not obtained (e.g. the data were analyzed anonymously)<br><br>**Animal Research (involving vertebrate animals, embryos or tissues)**<br>• Provide the name of the Institutional Animal Care and Use Committee (IACUC) or other relevant ethics board that reviewed the study protocol, and indicate whether they approved this research or granted a formal waiver of ethical approval<br>• Include an approval number if one was obtained<br>• If the study involved *non-human primates*, add *additional details* about animal welfare and steps taken to ameliorate suffering<br>• If anesthesia, euthanasia, or any kind of animal sacrifice is part of the study, include briefly which substances and/or methods were applied<br><br>**Field Research**<br><br>Include the following details if this study involves the collection of plant, animal, or other materials from a natural setting:<br>• Field permit number<br>• Name of the institution or relevant body that granted permission	
**Data Availability**<br><br>Authors are required to make all data underlying the findings described fully available, without restriction, and from the time of publication. PLOS allows rare exceptions to address legal and ethical concerns. See the PLOS Data Policy and FAQ for detailed information.	Yes - all data are fully available without restriction

A Data Availability Statement describing where the data can be found is required at submission. Your answers to this question constitute the Data Availability Statement and **will be published in the article**, if accepted.<br><br>**Important:** Stating 'data available on request from the author' is not sufficient. If your data are only available upon request, select 'No' for the first question and explain your exceptional situation in the text box.<br><br>Do the authors confirm that all data underlying the findings described in their manuscript are fully available without restriction?	
**Describe where the data may be found in full sentences. If you are copying our sample text, replace any instances of XXX with the appropriate details.**<br><br>• If the data are **held or will be held in a public repository**, include URLs, accession numbers or DOIs. If this information will only be available after acceptance, indicate this by ticking the box below. For example: *All XXX files are available from the XXX database (accession number(s) XXX, XXX.).*<br>• If the data are all contained **within the manuscript and/or Supporting Information files**, enter the following: *All relevant data are within the manuscript and its Supporting Information files.*<br>• If neither of these applies but you are able to provide **details of access elsewhere**, with or without limitations, please do so. For example:<br><br>*Data cannot be shared publicly because of [XXX]. Data are available from the XXX Institutional Data Access / Ethics Committee (contact via XXX) for researchers who meet the criteria for access to confidential data.*<br><br>*The data underlying the results presented in the study are available from (include the name of the third party*	The data underlying the results presented in the study are available from Korea Institute for Health Promotion and Development

and contact information or URL).<br>• This text is appropriate if the data are owned by a third party and authors do not have permission to share the data.<br><br>* typeset	
Additional data availability information:	Tick here if your circumstances are not covered by the questions above and you need the journal's help to make your data available.

Powered by Editorial Manager® and ProduXion Manager® from Aries Systems Corporation

author list
Click here to access/download;Manuscript;Plos one_author.docx

Analysis of related factors for adolescents' intention to use alcohol in Korea

Eun-A[1], Park, Ae-Ri[1], Jung, Sungyong, Choi[2]

[1] Departmeent of Nursing, Bucheon University, Bucheon, Korea

[2] Official, Big data division, Seoul Metropolitan Government, seoul, Korea

* Corresponding author

E-mail: aeri83@bc.ac.kr

These authors contributed equally to this work.

Funding

The authors received no financial support for the research, authorship, or publication of this article.

Author contribution

All authors contributed in study design, data analysis, and manuscript writing.

Conflict of interest

The authors declare no potential conflicts of interest with respect to the research, authorship, or publication of this article.

Manuscript Click here to access/download;Manuscript;Plos one_manuscript.docx

Abstract

The harmful effects of alcohol consumption by adolescents are increasingly emphasized. It is thus necessary to identify individual and environmental factors that encourage such drinking. This study investigated factors associated with the sustainable use of alcohol (SUA) in adolescents who consume alcohol, and the possibility of future drinking (PFD) in non-drinking adolescents. Data from 'the Adolescents Awareness Survey of Alcohol Encouraging Environment' by the Ministry of Health and Welfare and the Korea Health Promotion Institute (2017) were used. The survey was completed by 1038 subjects, selected through a proportional allocation extraction method, who were aged 13-18 years and lived in five cities with a population of over 1 million. The factors associated with SUA included gender (β=0.634, p<0.004), grade, drinking alone (β=-2.147, p<0.002), liquor advertising (β=1.644, p<0.0001), ease of alcohol purchase (β=1.541, p<0.025), parental permission to drink (β=1.084, p<0.0001), not knowing of mother's education level (β=-0.685, p<0.045), positive expectancies from drinking (β=0.141, p<0.0001), and the number of pubs (β=0.303, p<0.002), internet game cafes (β=0.456, p<0.019) and Karaokes (β=-0.098, p<0.023) in the community. The factors associated with the possibility of future drinking (PFD) in non-drinkers were grade, liquor advertising (β=-1.355, p<0.001), parental permission to drink (β=0.783, p<0.020), positive expectancies from drinking alcohol (β=0.139, p<0.0001) and the relationship with father (β=0.072, p<0.033). Multidimensional interventions, including those by individuals, parents, peers, and local communities, are needed therefore to prevent sustainable use of alcohol (SUA) and the possibility of future drinking (PFD) in adolescents.

Introduction

Alcohol is the most prevalent substance that adolescents abuse among harmful drugs such as cigarettes, drugs, and hallucinogenic substances.[1] According to?? The average age when adolescents initiated drinking alcohol was 13.2 years and the rate of alcohol use, defined as more than one drink before middle school, was 17.4% in 2018 and 16.0% in 2019.[2]

Previous studies have reported that adolescents who started drinking before the age of 15 years showed a higher risk of alcohol dependence and abuse,[3] alcohol-related car accidents,[4] and other unintended injuries[5] after adulthood than those who started drinking at age 21 or older. Further, since drugs such as tobacco and alcohol may act as gateways for illegal drugs, preventing alcohol consumption not only avoids or minimizes the harm caused by drinking alcohol, but also delays the use of other drugs and prevents drug-related harm.[6] Previous studies on the drinking behaviors of adolescents have found that the individual factors were related to positive attitudes toward drinking, including current smoking, knowledge about drinking and drugs, stress, depression, and positive drinking expectations that are aimed at improved sociability and sexual function .[7-8] The factors relating to the family and friends included the number of friends who drink, frequency of friends' drinking, and amount of alcohol that they consumed. The socio-demographic factors related to type of school, grade level, age, and gender.[9] In a study of adolescent problem drinking, their drinking motives, alcohol expectancies, depression, anxiety, and self-efficacy were found to be associated individual factors, and family factors were related to positive relationships and parental monitoring.[7] In particular, parental drinking or poor relationships with parents were factors associated with adolescents' alcohol use.[8-9] Parents' permission to drink in childhood displayed a significant association with an increased likelihood of risky drinking later in adolescence.[7] The main reasons for alcohol use were to maintain friendships, to talk openly with friends, to relieve stress, and because other friends drink.[10] This suggests that alcohol may be used as a medium to achieve social acceptance and status in peer groups. The positive expectancy of alcohol included relaxation from tension and stress relief.

Although Korea's central law prohibits the sale of alcohol to adolescents under the age of 19,[11] this was not a deterrent to alcohol consumption. Of the adolescents surveyed in 2019, 46.6% of middle school students and 73.6% of high school students found it easy to buy liquor

according to the 2019 Adolescent Health Behavior Survey.[2] It has been reported that non-drinkers are more likely to drink as their exposure to alcohol commercial advertising increases, as are current drinkers.[12] Exposure to liquor advertisements promotes adolescents' good impressions about alcohol use and encourages their intentions to use alcohol. It further influences adolescents who have never drunk alcohol to have a positive attitude and intention to use alcohol through the effective messaging of advertising.[13]

Adolescents with high levels of stress or those experiencing suicidal thoughts were at high risk of problem drinking(anyone who has experienced more than one post-drinking problem in the last 12 months).[14] Alcohol expectancies refer to the beliefs that a drinker has in the effects of drinking or the consequences of drinking, which is more symbolic than the actual alcohol consumption.[15] In a previous study, 33.0% of adolescents reported that their parents had recommended alcohol consumption.[16] Thus, adolescents often drink alcohol at the recommendation of their parents and other adults.

To summarized, this study in intended to provide the evidence for an adolescent drinking prevention policy by identifying factors that affect sustainable alcohol use (SUA), defined that they've had more than one drink in their life and they have the intension to use alcohol, and future drinking possibility (PFD), defined that they haven't had more than one drink in their life, but they have the intension to use alcohol in adolescents.

Materials and Methods

2.1. Participants

This descriptive cross-sectional study aimed to identify the factors influencing the SUA of alcohol-consuming adolescents and the PFD of non-alcohol-consuming adolescents.

The data for this study were obtained from the Environmental Drinking Survey of Adolescent Drinking Promotion conducted by the Ministry of Health and Welfare and the Korea Health Promotion Development Institute in 2017. The subjects for this survey were middle and high school students nationwide, aged 13 to 18 years, and included a total of 1,045.

Of these 1,045 adolescents, the data from 1,038 were analyzed, excluding: 1) two non-responders to the question on problem drinking, 2) one non-responder to the question on the intention to use alcohol, 3) one non-responder to the question on "felt like drinking alcohol after being exposed to advertisement"; 4) two non-responders to the question on "the last person I drank with", and 5) one non-responder to the question on attempting to purchase alcohol.

2.2. Instruments

2.2.1. Dependent variables

The dependent variable was the intention to use alcohol, which indicated the intention to use alcohol in the near future, and was measured using a tool developed by the University of Minnesota. We presented four situations: (1) when the age of 19 years (adult) is reached, (2) if someone recommends alcohol within a year, (3) if someone recommends alcohol within a month, and (4) if someone recommends alcohol within a week.

The intention to drink in each situation was measured using a 5-point Likert scale (from 'never' to 'very much'). The higher the score, the higher the possibility of drinking, and the Cronbach's α in this study was .81. An adolescent who drank more than one alcoholic drink in his or her life was defined as an adolescent who drank alcohol (drinker) and the others were defined as non-drinkers. The higher the score of a drinker answering the question regarding the tendency to use alcohol, the higher the SUA. On the other hand, the higher the score of non-

drinkers responding to the question regarding the tendency to use alcohol, the higher their PFD in the near future.

2.2.2. Independent variables

The following variables were investigated and selected by referring to the variables that affect adolescent drinking behavior in previous studies.

Positive expectations for drinking used eight positive expectations' measures from the 16 categories of drinking scale. These were selected and reconstructed by Yoon[17] from the youth types (AEQ-A) of drinking-level surveys developed by Christiansen, Smith, Roehling, and Goldman. These were measured on a 5-point Likert scale, and the higher the score, the higher the positive drinking range. The Cronbach's alpha in this study was .604.

The relationship between parents and children was assessed by asking eight questions and measured using a 4-point scale (from 'never' to 'very much'). The higher the score, the better the relationship between parents. Cronbach's α in this study was .962 (father), and .943 (mother).

Drinking variables were related to parents and peers. These included peers who drank alcohol among close friends, parents' permission to drink, relationship with parents, education level of parents, and the school's disciplinary action for drinking.

The motivation for drinking as promoted by the media was measured by "whether you ever wanted to drink alcohol after watching an advertisement for alcohol, an image of alcohol, or a drinking scene" (a single question).

Environmental variables measured the perceptions of drinking regulations, including punishment for students who drank alcohol at schools, and drinking experiences during school events such as travel and clubs (a single question). We confirmed whether the local governments had drinking ordinances in 2017 by searching the municipal regulations

information system.[18] The data were derived from the statistics of businesses related to alcohol consumption by the administrative district units; These administrative districts are the locations of schools where participants attend. The number of supermarkets (Korean Standard Industrial Classification Code: 47121), convenience store chains (47122), general retail (47129), general entertainment bars (56211), dance bars (56212), pubs (56213), other bars (56219), internet game cafes (91222), and Karaokes (91223) were analyzed.

Variables such as gender, grade, onset of drinking, ease of alcohol purchase, problem drinking, stress, and parents' education level were based on the Korea Youth Risk Behavior (KYRBS) Web-based Survey (http://yhs.cdc.go.kr). The KYRBS was established in 2005 by the Centers for Disease Control and Prevention in Korea. The KYRBS is an ongoing national cross-sectional survey that assesses health-risk behaviors among middle- and high-school students and monitors progress toward achieving the national health objectives of Korea's National Health Plan 2020 and 2030.

2.3. Procedures

This survey was conducted from October 30, 2017 to December 14, 2017 and targeting male and female adolescents aged 13-18 years who live in cities with a population of more than 1 million. Based on the results of the 2016 Basic Education Statistics Survey, adolescents were selected according to the stratified system extraction method. Considering that the subjects of the survey were adolescents, a professional interviewer conducted a face-to-face survey under the guidance of the school teacher only after obtaining consent from adolescents and parents. The community data were obtained from the 'Census of Establishment' conducted by Statistics Korea which investigated all businesses with one or more employees.

2.4. Data analysis

All statistical analyses were carried out using SAS software (version 9.4), and results with $p < .05$ were interpreted as being statistically significant. Variables influencing SUA in drinking adolescents and PFD in non-drinking adolescents were classified into individual variables, family and peer variables, and environmental variables.

First, the chi square test, t-test, and ANOVA tests were performed depending on the variable, to verify the differences between alcohol drinking and non-drinking adolescents. A descriptive analysis of the community circumstances' variables was performed.

The variables included thus were a) individual (sociodemographic, stress, intention to use alcohol, alcohol expectancy, ease of buying liquor, felt like drinking alcohol after being exposed to advertisement, etc.), b) peers (people I drank with lately), parents (relationship between parents and children), parental education level, parental permission to drink, etc.), and c) school/community circumstances (drinking experience at school events, recognized school drinking regulations for their experience, existence of local drinking ordinance or not, and the number of bars including supermarkets and convenience stores).

Linear regression were performed for exploring variables affecting the intention to use alcohol of adolescents to explore the relationship of the variables, a correlation the dependent variables were intention to use alcohol, with a continuous answer. The following procedure was used to estimate the internal consistency of the scores to examine the reliability of the instruments used for data collection.

Finally, hierarchical multiple regression was performed considering individual, parent, peer, and local community environmental variables simultaneously. This analysis method was used for separating individual and environmental factors and looking at their respective impacts.

This means that the relationship between the variables continues even if the analysis variables differ at the individual and community levels(the number of supermarkets, etc.).

2.5. Ethical consideration

The questionnaires and agreements developed for the survey of the youth drinking-control environment were reviewed by the Bioethics Committee of the Korea Institute for Health Promotion and Development (IRB 1709-HR-010-01). At the time, the investigation was conducted with the consent of parents, and the investigation was conducted with the guidance of the school teacher only if the consent of parents and adolescents was obtained.

Results

3.1. Characteristics of participants

From a comparative analysis of drinking behavior between drinking and non-drinking adolescents, there are shown in Table 1.

There was a significant difference in alcohol consumption between 628 (60.5%) and 410 non-drinkers (39.5%). There were 604 male (63.38%) and 434 female (36.62%) students. By grade, 238 students were in 10th grade, 195 in 11th grade, 183 in 8th grade, and 162 in 7th grade.

Initiation of alcohol consumption: There were 257 (40.92%) adolescents who started drinking before entering middle school (7th grade) and more than 13.0% had started by the 7th and 8th grades (p <0.0001).

Alcohol promoters: The most recent drinkers were those encouraged by family members, 355 (56.53%), followed by 228 (36.31%) encouraged by friends and classmate (p <0.0001)..

Problem drinking: Among the 628 drinkers, a total of 102 (16.24%) adolescents reported that they had problematic drinking (*p*<0.0001).What measure was used for harmful drinking?

Subject Stress: 184 (29.3%) of 628 drinkers were reported a lot of stress, as did 186 (45.37%) non-drinkers. "Middle" stress was reported by 262 drinkers (41.72%) and 146 non-drinkers (35.61%) (*p*<0.0001).

Liquor advertising: The idea of wanting to drink alcohol after encountering a liquor advertisement was especially noted among non-drinkers, with 393 drinkers (62.58%) and 333 non-drinkers (81.22%) encouraged to drink by advertisements (*p*<0.0001).

Ease of alcohol purchase: 134 (21.34%) of 628 drinker were reported that it was possible to buy alcohol, and 3(0.73%) of 410 non-drinking. In the case of alcohol purchases 'being impossible', 29 (4.62%) drinkers and 10 (2.44%) non-drinker (*p*<0.0001).

Positive expectations: The mean score for the positive expectations of the effect from drinking alcohol was 22.05 (±7.47) points for drinkers and 17.84 (±8.24) for non-drinkers, showing the high expectations for a drinking effect in the drinking group(*p*<0.0001). When asked about sustainable drinking or intention to use alcohol, drinkers (mean score 11.56 [±3.91] and non-drinkers (mean score 8.31 [±3.04]) showed significant differences(*p*<0.0001).

Table 1. Characteristics and differences between drinkers and non-drinkers in adolescents (N=1038)

Variables	Categories	Total	Drinkers	Non-drinkers	$X^2/t(p)$
			N (%) or Mean ± SD		
Gender	Male	604	398 (63.38)	206 (50.24)	17.582
	Female	434	230 (36.62)	204 (49.76)	(***)
Grade	7th grade	162	76 (12.1)	86 (20.98)	
	8th grade	183	91 (14.49)	92 (22.44)	
	9th grade	145	67 (10.67)	78 (19.02)	64.213
	10th grade	238	173 (27.55)	65 (15.85)	(***)
	11th grade	195	142 (22.61)	53 (12.93)	
	12th grade	115	79 (12.58)	36 (8.78)	

Onset of Drinking	No drink	410	0 (0)	410 (100)	1038 (***)
	Before entering middle school	257	257 (40.92)	0 (0)	
	7th grade	89	89 (14.17)	0 (0)	
	8th grade	84	84 (13.38)	0 (0)	
	9th grade	85	85 (13.54)	0 (0)	
	10th grade	85	85 (13.54)	0 (0)	
	11th grade	17	17 (2.71)	0 (0)	
	12th grade	11	11 (1.75)	0 (0)	
The last person I drank with	No drink	410	0 (0)	410 (100)	1038 (***)
	Friend or classmate	228	228 (36.31)	0 (0)	
	Senior or junior	15	15 (2.39)	0 (0)	
	Family or Relative	355	355 (56.53)	0 (0)	
	alone	25	25 (3.98)	0 (0)	
	Etc	5	5 (0.8)	0 (0)	
Problem drinking (n=188)	Yes	102	102 (54.26)	0 (0)	-
	No	86	86 (45.74)	0 (0)	
Subject stress level	None	260	182 (28.98)	78 (19.02)	30.136 (***)
	Middle	408	262 (41.72)	146 (35.61)	
	Many	370	184 (29.3)	186 (45.37)	
Felt to drink Alcohol after being exposured to advertisement	Not having	131	113 (17.99)	18 (4.39)	52.303 (***)
	Middle	181	122 (19.43)	59 (14.39)	
	Having	726	393 (62.58)	333 (81.22)	
Ease of alcohol purchase	Impossible	39	29 (4.62)	10 (2.44)	116.186 (***)
	Possible	137	134 (21.34)	3 (0.73)	
	Not know / no response	24	3 (0.48)	21 (5.12)	
	Not try	838	462 (73.57)	376 (91.71)	
Parent's permission to drink	No	622	305 (48.57)	317 (77.32)	98.188 (***)
	Yes	346	282 (44.9)	64 (15.61)	
	Not Applicable	70	41 (6.53)	29 (7.07)	
Scores of relationship with father		1038	25.92 ± 5.14	26.21 ± 5.46	-0.820 (0.414)
Scores of relationship with mother		1038	27.89 ± 4.57	28.2 ± 4.73	-1.030 (0.305)
Positive alcohol expectancy		1038	22.05 ± 7.47	17.84 ± 8.24	8.350

					(***)
Intention to use alcohol		1038	11.56 ± 3.91	8.31 ± 3.04	15.020 (***)
Father's level of education	≤Middle school	19	12 (1.91)	7 (1.71)	8.682 (0.070)
	High school	255	169 (26.91)	86 (20.98)	
	≥ College or university	483	290 (46.18)	193 (47.07)	
	Absence	32	22 (3.5)	10 (2.44)	
	Not know	249	135 (21.5)	114 (27.8)	
Mother's level of education	≤Middle school	20	13 (2.07)	7 (1.71)	22.032 (***)
	High school	309	218 (34.71)	91 (22.2)	
	≥ College or university	436	254 (40.45)	182 (44.39)	
	Absence	8	5 (0.8)	3 (0.73)	
	Not knowing	265	138 (21.97)	127 (30.98)	
Drinking experience during school events	No	936	526 (83.76)	410 (100)	65.632 (***)
	Yes	102	102 (16.24)	0 (0)	
Perception of drinking regulations	No	266	171 (27.23)	95 (23.17)	2.144 (0.143)
	Yes	772	457 (72.77)	315 (76.83)	

3.2. Environment that encourages drinking

The local drinking ordinance, the number of convenience store chains, general retail stores, general entertainment bars, dance bars, other bars, pubs, internet game cafes, and karaoke in 35 Gu(local) of five cities in Korea are shown in Table 2.

Local governments' drinking ordinances include prohibitions on drinking in public places such as parks, while 20 local governments have drinking-related ordinances although 15 do not. Among the number of places selling alcohol, there were 15.44 (±12.28) other bars per square kilometer followed by general retail stores, convenience store chains, and karaokes.

Table 2. Community Variables (N=35)

**Variables**	**Categories**	**N(%) or Mean ± SD**	***Min***	***Max***

Drinking ordinances of local governments	No	15 (57.1)	-	-
	Yes	20 (42.9)		
Supermarkets (per $1Km^2$)		1.75 ± 0.89	0.48	3.40
Convenience store chains (per $1Km^2$)		7.82 ± 7.06	1.51	33.73
General retail stores (per $1Km^2$)		8.73 ± 9.59	1.50	54.92
General entertainment bars (per $1Km^2$)		4.39 ± 3.36	0.72	18.07
Dance bars (per $1Km^2$)		0.26 ± 0.32	0.02	1.42
Pubs (per $1Km^2$)		1.93 ± 2.04	0.24	9.14
Other bars (per $1Km^2$)		15.44 ± 12.28	2.36	45.7
Internet game cafes (per $1Km^2$)		2.14 ± 1.39	0.40	6.15
Karaokes (per $1Km^2$)		7.79 ± 5.88	1.37	22.98

3.3. Factors influencing SUA in drinking adolescents

The significant variables affecting SUA for drinkers were gender, grade, drinking alone, and if they wanted to drink alcohol after watching liquor advertisements, parents' recommendations to drink alcohol, mother's level of education, and positive expectations for alcohol. The power of this explanation was 38.0% (Table 3).

Male teenagers' SUA scores were 0.683 points higher than that of the female students (p=0.012), and the 12th grade adolescents' SUA scores were 2.011 points higher than that of 7th grade students (p=0.000). Compared to those who drank alcohol with friends or classmates, SUA score was 2.215 points lower when drinking alone (p=0.003). Compared to "Middle", the idea of wanting to drink alcohol after encountering a liquor advertisement increased by 1.765 points (p<0.0001). SUA increased by 1.172 points when there were parents' recommendations to drink (p<0.0001). It was reduced by 0.711 points if adolescents did not know their mother's level of education, compared to those who knew that their mother graduated from college or had a higher level of education (p=0.037). SUA increased by 0.143 points when the expectations for positive drinking increased by 1 (p<0.0001).

3.4. Factors influencing PFD in non-drinking adolescents

The variables that affect PFD of non-drinking adolescents were grade, the desire to drink alcohol after watching liquor advertisements, parents' recommendation to drink alcohol, the degree of relationship with their fathers, and positive expectations for alcohol. The power of the explanation was 30.3% (Table 3).

By grade, PFD in 12th grade was 1.9 points higher than in 7th grade, which is considered to be twice as high as other grades, as the legal age of drinking alcohol is 19 years old. The desire to drink alcohol after watching liquor advertisements decreased by 1.337 points (p=0.001) compared to 'middle' and by 1.477 points (p=0.045) when responding 'having'. It increased by 0.815 points when there was no parental recommendation to drink (p=0.031). The stronger the relationship with their father, the higher PFD, which increased by 0.139 points (p<0.0001), and PFD was reduced by 0.094 points (p=0.035) when the expectation for positive drinking increased by 1.

3.5. Factors associated with SUA by hierarchical multiple regression

The variables for the drinking youth SUA were significant in respect of gender, grade, drinking alone, alcohol advertising, ease of alcohol purchase, parental alcohol recommendation, mother’s level of education, positive expectations for alcohol, local community pubs, internet game cafes and karaokes (Table 4).

SUA of male teenagers was 0.634 points higher than that of female drinkers (p=0.004), and this increased as the school grades increased. Compared to recent drinking with friends and alumni, drinking alone decreased by 2.147 points (p=0.002). The number of people who

wanted to drink after watching a commercial liquor advertisement increased by 1.644 points compared to "Middle" (p<0.0001). SUA was 1.541 points higher among those who experienced it as "easy to buy liquor" compared to those who experienced it as "impossible to buy alcohol" (p=0.025), which suggests that the act of drinking will be strengthened further by the successful purchase of alcohol. It increased by 1.084 points when recommended by parents (p<0.0001) compared to when parents did not recommend that their children drink alcohol. Compared to cases in which the mother's educational level was higher than that of a college graduate, the number of respondents who responded 'do not know' for the mother's educational level decreased consumption by 0.685 points (p=0.045). For drinkers, SUA increased by 0.141 points when positive expectations for alcohol increased by 1 (p<0.0001). However, the rate of increase was not higher than that of non-drinking teenagers, which is believed to be due to the fact that they have already experienced the reality of expectations for alcohol by drinking alcohol. In the local community, when the number of liquor stores, etc., increased by 1 per unit area of 1 km^2, SUA increased by 0.303 points (p=0.002), and when the number of internet game cafe increased by 1 per unit area of 1 km^2, SUA increased by 0.456 (p=0.019). On the other hand, SUA decreased by 0.098 points when he number of karaoke increased by 1 per unit area of 1 km^2 (p=0.023).

3.6. Factors associated with PFD by hierarchical multiple regression

The variables associated with PFD of non-drinkers were grade, the idea of wanting to drink after watching a liquor advertisement, parents' recommendation to drink alcohol, the relationship with their father, and positive expectations of alcohol (Table 4).

PFD increased amongst adolescents in the 8th grade (β=0.531, p=0.024), 10th grade (β=0.717, p=0.035), and 12th grade (β=1.882, p=0.001), when compared to the 7th grade. This is believed to increase the possibility of these adolescents drinking in the near future as 12th graders, equivalent to high school seniors, stress during college entrance exams, and Korea's drinking age, which allows drinking from 19 years or older, is imminent. The idea of drinking alcohol after watching a liquor advertisement was reduced by 1.355 points in PFD group when responding to 'Not having’ compared to 'Middle' (p=0.001). PFD increased by 0.783 points in the absence of parental drinking recommendations (p=0.020). PFD increased by 0.139 points, as positive expectations for alcohol increased by 1 (p<0.0001). As their relationship with their father increased by 1, PFD of adolescent increased by 0.072 points (p=0.033). On the other hand, the number of internet game cafes per 1 km^2, did not significantly affect PFD of non-drinkers.

Table 3. Factors Influencing Sustainable Use of Alcohol and Possibility of Future Drinking in Adolescents

Sustainable Use of in Drinking Adolescents						Possibility of Future Drinking in Non-drinking Adolescents					
**Variable**	**Ref**	**Categories**	**Parameter Estimate**	**Standard Error**	***t(p)***	**Variable**	**Ref**	**Categories**	**Parameter Estimate**	**Standard Error**	***t(p)***
Intercept			7.392	0.966	7.66 (<.001)	Intercept			6.949	1.094	6.35 (<.001)
Gender	Female	Male	0.683	0.271	2.52 (.012)						
Grade	7th grade	8th grade	0.659	0.497	1.33 (.185)	Grade	7th grade	8th grade	0.457	0.417	1.09 (.274)
		9th grade	0.750	0.540	1.39 (.165)			9th grade	0.315	0.430	0.73 (.465)
		10th grade	0.688	0.471	1.46 (.145)			10th grade	0.637	0.448	1.42 (.156)
		11th grade	0.870	0.490	1.78 (.076)			11th grade	0.738	0.529	1.39 (.164)
		12th grade	2.011	0.545	3.69 (<.001)			12th grade	1.900	0.563	3.38 (.001)
The last person I drank with	Friends or classmates	Senior or junior	-0.641	0.858	-0.75 (.455)	Subject stress level	Middle	Much	0.759	0.390	1.95 (.052)
		Family or relative	-0.617	0.330	-1.87 (.062)						
		Alone	-2.125	0.699	-3.04 (.003)			little	0.033	0.304	0.11 (.914)
		Etc.	-0.526	1.431	-0.37 (.713)						
Felt to drink alcohol after being exposured to advertisement	Middle	Not having	-0.611	0.345	-1.77 (.077)	Felt to drink alcohol after being exposured to advertisement	Middle	Not having	-1.337	0.415	-3.22 (.001)
		Having	1.765	0.419	4.21 (<.001)			Having	-1.477	0.733	-2.01 (.045)
Ease of alcohol purchase	Impossible	Easy	1.264	0.676	1.87 (.062)						
		Not knowing or Not response	-0.957	1.922	-0.5 (.619)						
		No attempt	-0.009	0.609	-0.01 (.988)						
Parent's permission to drink	No	Yes	1.172	0.270	4.35 (<.001)	Parent's permission to drink	No	Yes	0.815	0.377	2.16 (.031)
		Not Applicable	0.684	0.535	1.28 (.201)			Not Applicable	-0.318	0.594	-0.54 (.592)

Mother's level of education	≥ Graduating from junior college	Absence	-1.757	1.459	-1.2 (.229)	Scores of relationship with father			0.139	0.018	7.64 (<.001)
		≤Middle school	-1.018	0.910	-1.12 (.264)						
		≤High school	-0.286	0.297	-0.96 (.337)	Scores of relationship with mother			0.073	0.039	1.85 (.065)
		Not knowing	-0.711	0.339	-2.09 (.037)						
Drinking experience during school events	No	Yes	-0.319	0.370	-0.86 (.390)						
Positive alcohol expectancy			0.143	0.020	7.06 (<.001)	Positive alcohol expectancy			-0.094	0.044	-2.12 (.035)
		R^2=.380						R^2=.303			

Table 4. Factors Influencing Sustainable Use of Alcohol in Drinking and Non-drinking Adolescents by Hierarchical Multiple Regression

**Drinking Adolescents**						**Non-drinking Adolescents**					
**Variables**	**Ref**	**Categories**	**Estimate**	**Standard Error**	**t(*p*)**	**Variables**	**Ref**	**Categories**	**Estimate**	**Standard Error**	**t(*p*)**
Intercept			6.042	0.816	7.40(<.001)	Intercept			6.553	1.560	4.20(<.001)
Gender	Female	Male	0.634	0.219	2.90(.004)						
Grade	7th grade	8th grade	1.591	0.304	5.23(<.001)	Grade	7th grade	8th grade	0.531	0.235	2.27(.024)
		9th grade	1.674	0.318	5.26(<.001)			9th grade	0.438	0.447	0.98(.329)
		10th grade	1.497	0.464	3.23(.001)			10th grade	0.717	0.339	2.11(.035)
		11th grade	1.041	0.359	2.90(.004)			11th grade	0.606	0.585	1.04(.300)
		12th grade	2.610	0.536	4.87(<.001)			12th grade	1.882	0.570	3.30(.001)
The last person I drank with		Senior or junior	-0.562	0.908	-0.62(.536)	Subject stress level	Midd	Much	0.807	0.447	1.80(.072)

	Friends or classmates	Family or relative	-0.604	0.316	-1.91(.057)		le				
		Alone	-2.147	0.694	-3.10(.002)			Little	0.054	0.240	0.22(.824)
		Etc	-0.769	1.532	-0.50(.616)						
Felt to drink alcohol after being exposured to advertisement	Middle	Not having	-0.622	0.340	-1.83(.068)	Felt to drink alcohol after being exposured to advertisement	Midd le	Not having	-1.355	0.384	-3.53(.001)
		Having	1.644	0.377	4.36(<.001)			Having	-1.459	0.841	-1.74(.084)
Ease of alcohol purchase	Impossible	Possible	1.541	0.686	2.25(.025)	Scores of relationship with father			0.072	0.034	2.15(.033)
		Not know/ no response	-0.377	0.920	-0.41(.682)	Scores of relationship with mother			-0.092	0.050	-1.85(.065)
		Not try	0.198	0.560	0.35(.724)						
Parent's permission to drink	No	Yes	1.084	0.220	4.93(<.001)	Parent's permission to drink	No	Yes	0.783	0.335	2.34(.020)
		Not Applicable	0.677	0.509	1.33(.184)			Not Applicable	-0.403	0.440	-0.92(.360)
Mother's level of education	≥ College or university	Absence	-1.994	1.083	-1.84(.066)						
		≤Middle school	-1.111	0.596	-1.87(.063)						
		High school	-0.370	0.285	-1.30(.194)						
		Not knowing	-0.685	0.341	-2.01(.045)						
Drinking experience during school events	No	Yes	-0.264	0.344	-0.77(.442)						
Positive alcohol expectancy			0.141	0.020	7.17(<.001)	Positive alcohol expectancy			0.139	0.023	6.17(<.001)
Internet game cafes (per 1Km^2)			0.456	0.194	2.35(.019)	Internet game cafes (per 1Km^2)			0.155	0.129	1.20(.229)
Pubs (per 1Km^2)			0.303	0.098	3.08(.002)						
Other bars (per 1Km^2)			-0.010	0.013	-0.78(.434)						
Karaokes (per 1Km^2)			-0.098	0.043	-2.29(.023)						
R^2=.395,ICC=.13						R^2=.314, ICC=.12					

Discussion

This study identified current SUA drinking and non-drinking adolescents with PFD, living in large cities, and analyzed the factors affecting SUA and PFD using the adolescents' individual circumstances and community environment factors. The analysis was performed using hierarchical multiple regression. The study results show that the factors affecting SUA in drinking adolescents are gender, grade, drinking alone, liquor advertising, ease of purchase of alcohol, parents' recommendation to drink alcohol, education level of mother, positive expectancies at the individual level and drinking alone. These were found to be negatively associated with SUA. The associated community factors were the number of bars, internet game cafes, and karaokes in the community environment. SUA explanatory power of these variables was 39.5%.

The factors related to the adolescents' individual circumstances that were associated with PFD in non-drinking adolescents were grade, liquor advertising, parents' recommendation to drink alcohol, positive expectancies of drinking, and the relationship with their father. The example of not thinking about drinking after watching an alcoholic beverage advertisement was found to have a negative association with PFD. There were no statistically significant variables for PFD associated with the community environment factors. PFD explanatory power of the above variables was 31.4%.

In this study, parents' recommendations to drink alcohol were found to increase drinking intentions among both drinking adolescents and non-drinking adolescents, which is consistent with the results of previous studies that reported that the more parents drink, the more their children experience drinking alcohol.[19]

In Korea, the experience of being encouraged to drink increases during family events.[20] This might be due to the social perception that adolescents can learn proper drinking etiquette from

adults. It shows a tolerant social climate toward alcohol and parents' recommendations to drink encouraged drinking adolescents, since it is interpreted that as tacit acceptance of drinking, thus alleviating the guilt about ignoring the ban on drinking under the age of 19. This suggests that the culture of tolerance to drinking in Korean society is deeply embedded in each family.

The parents' role is most important in preventing adolescent drinking, and parental restrictions are necessary, because strict parental control can lower the adolescent drinking rate.[16] Since adolescence is a period in which attitudes toward drinking are formed, it is necessary to change the false belief that parents' recommendations to drink alcohol will have a positive effect on improving the parental adolescent relationship or on the establishment of good drinking attitudes through accurate education on drinking.[21]

Among parent-child relationships, the relationship with the father was not significantly associated with alcohol consumption in drinking adolescents, but was found to be a weak influencing variable for PFD in non-drinking adolescents. This differs from the results of a previous study that reported reduced levels of alcohol use/misuse and delayed the initiation of drinking alcohol and such intentions when the parent-child relationship was good.[22] However, drinking and non-drinking adolescents are still required to understand drinking prevention education and that the rationale for this is to promote their health.

Alcohol advertising appeared to be associated with intentions to consume alcohol in both drinking and non-drinking adolescents. Drinking adolescents had increased SUA levels following exposure to alcohol advertising. However, for non-drinking adolescents, the PFD decreased by 1.355 points when they said they had no intention of drinking after watching an alcohol advertisement. This result was partially consistent with another result where the preference for alcohol advertising was high and the likelihood of drinking increased as exposure to advertising increased, with drinkers indicating that they drink more, and non-drinkers reporting a high intention to drink.[13,23] TV, mass media, print advertisements, and the

Internet social media, which can be easily accessed by young people, are also targets related to alcohol consumption. Positive emotional experiences, achievements, individuality, and camaraderie about drinking, observed through the media can influence the onset, duration, and frequency of drinking. Therefore, continuous monitoring and policy interventions in various media are necessary.

For drinking adolescents, the SUA increased by 0.141 points ($p<.001$) when positive alcohol expectancy increased by 1, but PFD decreased by 0.094 points for non-drinking adolescents. A positive alcohol expectancy is a strong predictor of SUA. In the case of drinking adolescents, it was consistent with previous studies that the higher the positive expectations about drinking, the higher the drinking continuity.[15] However, the opposite result was found for non-drinking adolescents. Positive alcohol expectancy has been reported to have a significant effect on the onset of alcohol consumption.[15] In the case of adolescent drinking, it is considered that the effect on SUA is not large because they have already experienced alcohol expectations in reality.

Among the factors related to individual circumstances, an association was found between drinking adolescents intentions to drink with friends or schoolmates compared to drinking alone. Previous studies on adolescents from ordinary families and multicultural families have identified drinking alone as a common risk factor.[24] It shows a different context from these study results in that drinking alone in other studies could increase the risk of drinking, by increasing the amount of drinking among adolescents.[25] On the other hand, if the reason for drinking alone in adolescents is simply the expectation that negative emotions can be reduced,[26] it is considered that this may not lead to SUA. Further research is needed to identify mediating factors in the future.

As a community environment factor associated with SUA in drinking adolescents, it was found the number of bars and internet game cafes. Internet use amongst adolescents is

positively related to drinking; the study is based on the evidence that exposure to alcohol through the Internet and social networking sites(SNS) increases, and the drinking rate increases through social online activities with friends.[27] The fact that there are many places for adolescents to meet and to buy alcohol in the community can be interpreted as the provision of an atmosphere in the community that is tolerant of drinking. Internet use also leads to decreased cognitive and behavioral self-control, which increases problematic drinking behavior.[28] This study found that the alcohol environment in the community was directly associated with adolescents' drinking intentions. In the case of an alcohol prevention policy, it is necessary to actively promote policies that lead to environmental control, such as the regulation of the number of pubs and places where alcohol is sold in the local community.

The current drinking prevention education in schools for adolescents is formal and fragmentary, but there has been no increase in the prevalence of reported adolescents' alcohol drinking. It will be necessary to develop a specific drinking prevention education program for drinking and non-drinking adolescents as well as active drinking education and individual counseling at every grade level. Early drinking in adolescence is an early drug abuse behavior that may lead to problematic drinking in adulthood, which also affects health behavior problems. Therefore, if there is no solution to the current drinking behavior of drinking adolescents, it may become a risk factor for future social problems. It is thus necessary to effectively deliver messages such as the application of the 'zero tolerance principle' to legal regulations related to drinking, such as the 'prohibition of drinking under the age of 19', along with alcohol education for drinking adolescents.

Conclusions

One of the goals of the adolescent drinking prevention policy is to delay the initiation of alcohol consumption by changing the legal drinking age. This is because the earlier the drinking age, the greater the risk of disability regarding adolescents' growth and development, and the higher the likelihood of substance abuse or addiction after adulthood. In this context, it is essential in order to prevent/reduce drinking among adolescents to identify factors related to their drinking intentions and to consider the possibility of drinking for non-drinkers who have not yet started drinking, especially adolescents who have experimented only once or twice out of curiosity.

This study is a cross-sectional study that aimed to examine the drinking behavior of adolescents, and the study had some limitations. Since this study has a cross-sectional design, it was not possible to prove the direction of the association. In addition, since only the variables included in this study were considered, a follow-up study considering other factors would be necessary.

References

1. Spear LP. Effects of adolescent alcohol consumption on the brain and behaviour. *Nature Rev Neurosci*. 2018;19(4):197–214. https://doi.org/10.1038/nrn.2018.10
2. Ministry of Health and Welfare, Korea Centers for Disease Control and Prevention[Internet]. The Korea Youth Risk Behavior Web-based Survey; 2019. Accessed November 10, 2020. http://www.kdca.go.kr/yhs/
3. Sartor CE, Lynskey MT, Heath AC, Jacob T, True W. The role of childhood risk factors in initiation of alcohol use and progression to alcohol dependence. *Addiction.* 2007;102(2):216-225. https://doi.org/10.1111/j.1360-0443.2006.01661.x
4. Hingson R, Edwards EM, Heeren T, Rosenbloom D. Age of drinking onset and injuries,

motor vehicle crashes, and physical fights after drinking and when not drinking. *Alcohol Clin Exp Res*. 2009;33:783-790. https://doi.org/10.1111/j.1530-0277.2009.00896.x

5. Hingson RW, Heeren T, Jamanka A, Howland J. Age of drinking onset and unintentional injury involvement after drinking. *Jama*. 2000, 284, 1527-1533. https://doi.org/10.1001/jama.284.12.1527
6. Kirby T, Barry AE. Alcohol as a Gateway Drug: A Study of US 12th Graders. *J of School Health*. 2012;82(3):371-379. https://doi.org/10.1111/j.1746-1561.2012.00712.x
7. Chung SK, Kim JS. A Meta-Analysis of Factors Related to Adolescent Problem Drinking. *Korean J Health Educ Promot*. 2014;31:71-83.
8. Sharmin S, Kypri K, Khanam M, Wadolowski M, Bruno R, Mattick RP. Parental supply of alcohol in childhood and risky drinking in adolescence: systematic review and meta-analysis. *Int J Environ Res Public Health*. 2017;14(3):287. https://doi.org/10.3390/ijerph14030287
9. Hummel A, Shelton K, Heron J, Heron J, Moore L, van den Bree MB. A systematic review of the relationships between family functioning, pubertal timing and adolescent substance use. *Addiction*. 2013;108(3):487–496. https://doi.org/10.1111/add.12055
10. JeKarl J, Heo YH, Kim SH. A Study on the Characteristics of Drunk Youth and its Improvement Plan. Korea: Committee on Youth Protection; 2004.
11. Ministry of Government Legislation web site Korea. Accessed November 10, 2020. https://www.moleg.go
12. Anderson P, de Bruijn A, Angus K, Gordon R, Hastings G. Impact of Alcohol Advertising and Media Exposure on Adolescent Alcohol Use: A Systematic Review of Longitudinal Studies. *Alcohol Alcohol*. 2009;44(3):229–243. https://doi.org/10.1093/alcalc/agn115
13. Scott S, Muirhead C, Shucksmith J, Tyrrell R, Kaner E. Does industry-driven alcohol marketing influence adolescent drinking behaviour? A systematic review. *Alcohol Alcohol*. 2017;52(1):84-94. https://doi.org/10.1093/alcalc/agw085

14. Lee H, Park S. Patterns of drinking behaviors and predictors of class membership among adolescents in the Republic of Korea: a latent class analysis. *J Korean Acad Nurs*. 2019;49(6):701-12.

15. Smit K, Voogt C, Hiemstra M, Kleinjan M, Otten R, Kuntsche E. Development of alcohol expectancies and early alcohol use in children and adolescents: A systematic review. Clinical psychology review. 2018;60:136-46.

16. Østergaard J, Järvinen M, Andreasen AG. A matter of rules? A longitudinal study of parents' influence on young people's drinking trajectories. *Eur Addict Res*. 2018;24(4):206-15. https://doi.org/10.1159/000492323

17. Yoon HM, Kim YS, Jang SO. Alcohol expectancies in relation to their drinking practices among Korean high school students. *Korean Journal of Social Welfare*. 1999;38:153-79.

18. Municipal regulations information system web site KOR. Accessed October 22, 2020. http://elis.go.kr

19. Randolph KA, Cheatham LP, Weiss UK, Williams J. Exposure to parent and peer alcohol use and the risk of drinking onset and escalation among adolescents. *Child Adolesc Social Work J*. 2018;35(2):97-106. https://doi.org/10.1007/s10560-017-0516-2

20. JeKarl J, Kim KK, Choi MJ. Intention to use alcohol among adolescents without having experience of alcohol use: Using the theory of planned behavior. *Korean J Health Educ Promot* . 2019;36:1-14. https://doi.org/10.14367/kjhep.2049.36.3.1

21. Donovan JE. Really underage drinkers: The epidemiology of children's alcohol use in the United States. *Prevention Science*. 2007;8(3):192-205. https://doi.org/10.1007/s11121-007-0072-7

22. Yap MB, Cheong TW, Zaravinos- Tsakos F, Lubman DI, Jorm AF. Modifiable parenting factors associated with adolescent alcohol misuse: a systematic review and meta- analysis of longitudinal studies. *Addiction*. 2017;112(7):1142-62. https://doi.org/10.1111/add.13785

23. Sharp CA, Bellis MA, Hughes K, Ford K, Di Lemma LC. Public acceptability of public health policy to improve population health: A population- based survey. *Heal Expect*. 2020;23(4):802-812. https://doi.org/10.1111/hex.13041

24. Park S. Substance use and sexual behaviors of adolescents in multicultural families in Korea. *Psychiatry investigation.* 2015;12(4):466. https://doi.org/10.17924/solc.2019.54.161

25. Creswell KG, Chung T, Clark DB, Martin CS. Solitary alcohol use in teens is associated with drinking in response to negative affect and predicts alcohol problems in young adulthood. *Clin Psychol Sci*. 2014;2(5):602-10.

26. Tucker JS, Ellickson PL, Orlando M, Martino SC, Klein DJ. Substance use trajectories from early adolescence to emerging adulthood: A comparison of smoking, binge drinking, and marijuana use. *J Drug Issues*. 2005;35(2):307-32.

27. Svensson R, Johnson B. Internet use and adolescent drinking: Does it matter what young people do online? *Drug Alcohol Depend*. 2020;213:108138. https://doi.org/10.1016/j.drugalcdep.2020.108138

28. Gámez-Guadix M, Calvete E, Orue I, Las Hayas C. Problematic Internet use and problematic alcohol use from the cognitive–behavioral model: A longitudinal study among adolescents. *Addict Behav*. 2015;40:109-14.
