# Supplementary material for: Analysis of related factors for adolescents’ intention to use alcohol in Korea
Source: PLoS One. 2022 Oct 11;17(10):e0275957. doi: 10.1371/journal.pone.0275957 (PMC9553045; doi:10.1371/journal.pone.0275957)
Supplement: Attachment — Submitted filename: Review_Plos One_16 Nov 2021.docx [file pone.0275957.s002.docx]

Review – Plos One – 16 Nov 2021

1. The introduction is framed in a way that indicates that this study is redundant as the introduction points out existence of the very evidence that this paper reports about. The authors will do well to highlight the gaps in research that this study addresses.
2. The content of the paper is overwhelming given the numerous variables included. The authors would do well to present a (clearer) conceptual or theoretical framework that more clearly explains the inclusion of all these variables. They already allude to it in pointing out that factors that contribute to adolescent alcohol use are layered from individual level to community level.
3. The univariate results presented under 3.3 and 3.4 (which I gather are a check for significant predictors to be included in the hierarchical regression model) do not need to be discussed in that much detail. The authors can simply indicate which variables were significant and which were not in the findings/results section, and completely leave them out of the discussion section.
4. It seems that the authors are presenting the results of table 2 as though an association was tested between the drinking venues density variables and the dependent variables. The authors need to present results of an association if indeed they hope to draw such conclusions.
5. The manuscript needs thorough editing:
   a. “Previous studies on the drinking behaviors of adolescents have found that the individual factors were related to positive attitudes toward drinking, including current smoking, …” do the authors mean substance use, given the next part of the sentence?
   b. “On the other hand, the higher the score of nondrinkers responding to the question regarding the tendency to use alcohol, ...” do the authors mean intention?
   c. “Positive expectations for drinking used eight positive expectations’ measures from the 16 categories of drinking scale.” This sentence is not so clear.
   d. “This is because the earlier the drinking age, the greater the risk of disability regarding adolescents’ growth and development” – alcohol-related disability?
6. “Alcohol is the *most prevalent* substance that adolescents abuse among harmful drugs such as cigarettes, drugs, and hallucinogenic substances” – do the authors mean ‘one of’ rather than ‘the most’? It would also be useful for the authors to indicate whether these are global or national patterns.
7. “The dependent variable was the intention to use alcohol, which indicated the intention to use alcohol in the near future, and was measured using a tool developed by the University of Minnesota” More information about the tool is needed. What is its name, when was it developed, what are its psychometric properties of the tool, how are the questions phrased, how it is scored, what is the full reference of the source? The authors say they presented four situations - are these 4 selected from x, if so from how many, and why these 4?
8. “The *following procedure* was used to estimate the internal consistency of the scores to examine the reliability of the instruments used for data collection.” Which following procedure?
9. “There was a significant difference in alcohol consumption between 628 (60.5%) and 410 non-drinkers (39.5%).” What do the authors mean by alcohol consumption here?
10. Table 1, rows 3 and 4 – the figures in the p-value column do not seem to be p-values (possibly sample size). I would also suggest that the chiquare and t-test results be presented in turn rather than mixed as they currently are in the table.
11. A few points in the discussion are misaligned with the findings and/or need further engagement:
    a. “However, drinking and non-drinking adolescents are still required to understand drinking prevention education and that the rationale for this is to promote their health.” - This conclusion appears unrelated to the authors’ summary of the findings in this paragraph.
    b. How do the authors explain the negative association between positive expectations and low likelihood of drinking among non-drinking adolescents?
    c. “Among the factors related to individual circumstances, an association was found between drinking adolescents intentions to drink with friends or schoolmates compared to drinking alone.” – this is confusing as there is reference to an association as well as a comparison. I suggest the authors rephrase this and be sure to present in a way that is supported by the research question and findings.
    d. “This study found that the alcohol environment in the community was directly associated with adolescents’ drinking intentions.” – as indicated earlier, there is no evidence of this association from the findings that are currently presented.
    e. “In addition, since only the variables included in this study were considered, a follow-up study considering other factors would be necessary.” – this statement is quite broad, it would be useful for the authors to provide more specific detail. What do the authors think is missing in understanding drinking intentions among adolescents?

Other general comments

I wonder whether the authors may consider using the term participants as it has become a more preferred term than subjects in the research field.

“In the case of drinking adolescents, it was consistent with previous studies that the higher the positive expectations about drinking, the higher the *drinking continuity*.” – sustained drinking and/or future drinking? Consistency in use of terms is needed and this needs to be checked throughout the manuscript.

References are needed for these statements:

“Local governments' drinking ordinances include prohibitions on drinking in public places such as parks, while 20 local governments have drinking-related ordinances although 15 do not.” – a reference is needed for this statement.

“Positive emotional experiences, achievements, individuality, and camaraderie about drinking, observed through the media can influence the onset, duration, and frequency of drinking.”
