# Supplementary material for: Analysis of related factors for adolescents’ intention to use alcohol in Korea
Source: PLoS One. 2022 Oct 11;17(10):e0275957. doi: 10.1371/journal.pone.0275957 (PMC9553045; doi:10.1371/journal.pone.0275957)
Supplement: Attachment — Submitted filename: Plos one_Response to Reviwes.docx [file pone.0275957.s003.docx]

Journal Requirements

| 1. Please ensure that your manuscript meets PLOS ONE's style requirements, including those for file naming. | We checked PLOS ONE's style requirements. |
| --- | --- |
| 2. Please amend your current ethics statement to address the following concerns | We agree with this suggestion. We revised on parts of 2.5. Ethical consideration. |
| 3. Please include additional information regarding the survey or questionnaire used in the study and ensure that you have provided sufficient details that others could replicate the analyses. | We agree with this suggestion. We added additional information regarding the survey on parts of 2.2. instruments.  The questionnaire and data of this study were created by the Korea Health Promotion Institute using the original materials of various tools and can be received at the request of the Korean information system site (<https://www.open.go.kr)> |
| 4. In your Data Availability statement, you have not specified where the minimal data set underlying the results described in your manuscript can be found. | We agree with this suggestion. This is a Korean information system site ([https://www.open.go.kr/](https://www.open.go.kr/rqestMlrd/rqestReg/rqestRegWritingForm.do)), and anyone who needs data can request data through information disclosure. Data can be provided within 10 working days upon request. |

Editor comment

| 1. It would be helpful to ensure consistency in presenting the p values (3 decimal points) | We agree with this suggestion. We revised presenting the p values (3 decimal points). |
| --- | --- |
| 2. Consider presenting the 95% confidence intervals. | We agree with this suggestion. So based upon the suggestion, we presented the opinions in the table 3 & 4. |
| 3. Make to detail whether the analysis was adjusted for the survey design (sampling weights, stratification and clustering). | We agree with this suggestion. So based upon the suggestion, we have revised on parts of 2.4. Data analysis. In this study, clustering was performed by school type (middle school, high school, special high school, special purpose high school) and didn’t set the sampling weights. |

Reviewer #1.

| The manuscript would benefit from a complete grammar and spell check. | We agree with this suggestion. We completed grammar and spell check. |
| --- | --- |
| Some key measures reported e.g. Problem drinking and Stress have not been adequately explained. | We agree with this suggestion. So based upon the suggestion, We have revised on parts of 2.2. Instruments. |
| the background needs to highlight the issue of underage drinking more strongly in order to build an argument and strengthen the justification for this study. | We agree with this suggestion. So based upon the suggestion, We have revised on parts of introduction in order to build an argument and strengthen the justification. |
| The manuscript would have benefited from a conceptual framework e.g. ecological model to explain the multiple influences on adolescent drinking or intention to drink. | We agree with this suggestion. So based upon the suggestion, We added conceptual framework on fig. 1. (Page 8) |

Reviewer #2.

| 1. The introduction is framed in a way that indicates that this study is redundant as the introduction points out existence of the very evidence that this paper reports about. The authors will do well to highlight the gaps in research that this study addresses. | We agree with this suggestion. So based upon the suggestion, we have revised line 30 - 62 on parts of introduction. Introduction is highlighted red font in this revised manuscription. |
| --- | --- |
| 2. The content of the paper is overwhelming given the numerous variables included. The authors would do well to present a (clearer) conceptual or theoretical framework that more clearly explains the inclusion of all these variables. They already allude to it in pointing out that factors that contribute to adolescent alcohol use are layered from individual level to community level. | We agree with this suggestion. So based upon the suggestion, we have revised line 80 - 94 on parts of materials and methods. Framework of study have added in line 132. |
| 3. The univariate results presented under 3.3 and 3.4 (which I gather are a check for significant predictors to be included in the hierarchical regression model) do not need to be discussed in that much detail. The authors can simply indicate which variables were significant and which were not in the findings/results section, and completely leave them out of the discussion section. | We agree with this suggestion. So based upon the suggestion, we have deleted the detailed description of the results of the text on the parts of 3.3., 3.4. and discussion. |
| 5. The manuscript needs thorough editing: a. “Previous studies on the drinking behaviors of adolescents have found that the individual factors were related to positive attitudes toward drinking, including current smoking, …” do the authors mean substance use (rather than drinking), given the next part of the sentence? b. “On the other hand, the higher the score of nondrinkers responding to the question regarding the tendency to use alcohol, ...” do the authors mean intention rather than tendency? c. “Positive expectations for drinking used eight positive expectations’ measures from the 16 categories of drinking scale.” This sentence is currently not so clear. d. “This is because the earlier the drinking age, the greater the risk of disability regarding adolescents’ growth and development” – alcohol-related disability rather than any disability? | We agree with this suggestion. So based upon the suggestion,   a. We have revised line 30 - 35 on parts of Introduction. We checked the references again and revised them to fit the context b. We have revised line 95 - 97 on parts of introduction. We changed "tendency" to "intention" to suit our intentions. c. We have revised line 102 - 106 on parts of independent variables. d. We have revised line 385 on parts of conclusions |
| 6. “Alcohol is the most prevalent substance that adolescents abuse among harmful drugs such as cigarettes, drugs, and hallucinogenic substances” – do the authors mean ‘one of’ rather than ‘the most’? It would also be useful for the authors to indicate whether these are global or national patterns. | We agree with this suggestion. So based upon the suggestion, we have revised line 22 - 23 on parts of Introduction |
| 7. “The dependent variable was the intention to use alcohol, which indicated the intention to use alcohol in the near future, and was measured using a tool developed by the University of Minnesota” More information about the tool is needed. What is its name, when was it developed, what are its psychometric properties of the tool, how are the questions phrased, how it is scored, what is the full reference of the source? The authors say they presented four situations - are these 4 selected from x, if so from how many, and why these 4? | We agree with this suggestion. So based upon the suggestion, We have added and revised line 80 - 94 on parts of 2.2.1 dependent variables. |
| 8. “The following procedure was used to estimate the internal consistency of the scores to examine the reliability of the instruments used for data collection.” Which following procedure? | We agree with this suggestion. So based upon the suggestion, we have deleted this sentence because it didn't fit the context. |
| 9. “There was a significant difference in alcohol consumption between 628 (60.5%) and 410 non-drinkers (39.5%).” What do the authors mean by alcohol consumption here? Also, 'drinkers' seems to be missing. | We agree with this suggestion. So based upon the suggestion, we have added line 188 on parts of 3.1. characteristics of participants. |
| 10. Table 1, rows 3 and 4 – the figures in the p-value column do not seem to be p-values (possibly sample size). I would also suggest that the Chisquare and t-test results be presented in turn rather than mixed as they are now in the table. | We agree with this suggestion. So based upon the suggestion,   a. We reviewed the results in Table 1. According to the results of the analysis, the current figure 1038 is the correct value. b. We presented the results of additional analysis of chi-square, t-test in the table1. |
| 11. A few points in the discussion are misaligned with the findings and/or need further engagement: a. “However, drinking and non-drinking adolescents are still required to understand drinking prevention education and that the rationale for this is to promote their health.” - This conclusion appears unrelated to the authors’ summary of the findings in this paragraph. b. How do the authors explain the negative association between positive expectations and low likelihood of drinking among non-drinking adolescents? c. “Among the factors related to individual circumstances, an association was found between drinking adolescents intentions to drink with friends or schoolmates compared to drinking alone.” – this is confusing as there is reference to an association as well as a comparison. I suggest the authors rephrase this and be sure to present in a way that is supported by the research question and findings. d. “This study found that the alcohol environment in the community was directly associated with adolescents’ drinking intentions.” – as indicated earlier, there is no evidence of this association from the findings that are currently presented. e. “In addition, since only the variables included in this study were considered, a follow-up study considering other factors would be necessary.” – this statement is quite broad, it would be useful for the authors to provide more specific detail. What do the authors think is missing in understanding drinking intentions among adolescents? | We agree with this suggestion. So based upon the suggestion   a. We deleted this part because it was not relevant. b. We have revised line 344-351 on parts of discussion. c. We have revised line 352-356 on parts of discussion.   d. We added this association from the findings on parts of result (3.2. Environment that encourages drinking).   e. We have revised line 392-394 on parts of conclusion. |

General comment

| Other general comments I wonder whether the authors may consider using the term participants as it has become a more preferred term than subjects in the research field. | We agree with this suggestion. So based upon the suggestion, we changed this term (subjects -> participants). |
| --- | --- |
| “In the case of drinking adolescents, it was consistent with previous studies that the higher the positive expectations about drinking, the higher the drinking continuity.” – sustained drinking and/or future drinking? Consistency in use of terms is needed and this needs to be checked throughout the manuscript. | We agree with this suggestion. So based upon the suggestion, we revised sustained drinking and checked throughout the manuscript. |
