# Supplementary material for: Analysis of related factors for adolescents’ intention to use alcohol in Korea
Source: PLoS One. 2022 Oct 11;17(10):e0275957. doi: 10.1371/journal.pone.0275957 (PMC9553045; doi:10.1371/journal.pone.0275957)
Supplement: Attachment — Submitted filename: Plos one review2.docx [file pone.0275957.s005.docx]

Plos one review

- Above abstract says “sex” Abstract in main text says “gender”- choose on term and keep consistent throughout the manuscript
- Remove d from the word “summarized” to read “summarize”
- Remove “in” before the word “intended”
- Change “they’ve” to “they have”- colloquial
- Change “have’nt” to have not- colloquial
- Correct spelling of “intension” to “intention”

**Independent variables**

- When describing the variables e.g. relationship between parents and children, provide an at least 1-2 examples of questions asked.

**Characteristics of sample**

- Remove .. after “Alcohol promoters: The most recent drinkers were those encouraged by family members, 355 (56.53%), followed by 228 (36.31%) encouraged by friends and classmate (p <0.0001)..”
- Subject Stress:……………”a lot of stress” -explain what is “a lot of stress”

**Table 1**

- Explain with a footnote what (***) mean

**Table 3:**

- Check grammar on “Felt to drink after exposured to alcohol” Should read something like “Felt like drinking after being exposed to alcohol”
- Unclear what “scores of relationship with father” measured= please explain
