# Supplementary material for: Analysis of related factors for adolescents’ intention to use alcohol in Korea
Source: PLoS One. 2022 Oct 11;17(10):e0275957. doi: 10.1371/journal.pone.0275957 (PMC9553045; doi:10.1371/journal.pone.0275957)
Supplement: Attachment — Submitted filename: Response to Reviewers_V3.docx [file pone.0275957.s006.docx]

| Editor comment | Answer |
| --- | --- |
| - Above abstract says “sex” Abstract in main text says “gender”- choose on term and keep consistent throughout the manuscript - Remove d from the word “summarized” to read “summarize” - Remove “in” before the word “intended” - Change “they’ve” to “they have”- colloquial - Change “have’nt” to have not- colloquial - Correct spelling of “intension” to “intention” | We agree with this suggestion. So based upon the suggestion,   - We revised on this term (gender) and keep consistent. - We checked and changed the grammar. |
| **Independent variables**   - When describing the variables e.g. relationship between parents and children, provide an at least 1-2 examples of questions asked. | We agree with this suggestion. So based upon the suggestion, We have added on parts of 2.2.2. independent variables. |
| **Characteristics of sample**   - Remove .. after “Alcohol promoters: The most recent drinkers were those encouraged by family members, 355 (56.53%), followed by 228 (36.31%) encouraged by friends and classmate (p <0.0001)..” - Subject Stress:……………”a lot of stress” -explain what is “a lot of stress” | We agree with this suggestion. So based upon the suggestion,   - We have deleted line 203-205. - We have revised “ a lot of stress” to a high level of stress [line 208] and table1 |
| **Table 1**   - Explain with a footnote what (***) mean | We agree with this suggestion. So based upon the suggestion, we changed this mark.  (*** -> *p*<0.001) |
| **Table 3:**   - Check grammar on “Felt to drink after exposured to alcohol” Should read something like “Felt like drinking after being exposed to alcohol” - Unclear what “scores of relationship with father” measured= please explain | We agree with this suggestion,   - We checked and changed the grammar. - We revised ‘The degree of close relationship with father’ |
