# Supplementary material for: Analysis of related factors for adolescents’ intention to use alcohol in Korea
Source: PLoS One. 2022 Oct 11;17(10):e0275957. doi: 10.1371/journal.pone.0275957 (PMC9553045; doi:10.1371/journal.pone.0275957)
Supplement: Attachment — Submitted filename: Review Plos One round 2.docx [file pone.0275957.s007.docx]

Review Plos One (round 2)

- Change “liquor commercial” to either “liquor commercials” or “exposure to liquor commercials”
- Please use the word “participant” to describe “subjects”, and ensure “participants” is used consistently throughout the manuscript.
- Line 41 change recommendation to “drinking” to recommendations to “drink” in childhood.
- Line 96, what does “were revised to 19 years old” mean? Do the authors means revised for use with 19 year olds?
- Line 125 change parents recommendation to “drinking” to recommendations to “drink”
- Line 141- change “Karaokes” to “karaokes”
- Line 144- change “subject” to “participant”
- Line 159- add “people” after 1 million.
- Line 162- change coeducational to “co-educational”
- The framework for the study is a useful addition. However, I suggest that authors refer to the framework earlier on when discussing the factors at the individual, family and school levels, Inserting a prompt such as (See Figure 1) intermittently in the Line 110, 115, 123, 143 helps the reader to see the relationship between the framework and the factors discussed under the heading independent variables.
- Line 166- Among the class participants “to”- change to “in” the survey,
- Line 166- What do the authors means by “special arrangements” were excluded. This needs to be explained.
- Line 183- change “subject” to “participant”
- Line 185- change “drinking” to “drink”
- Same with Line 187
- Section 25: Ethical considerations- Please add details on how the data was stored, was it anonymised what measures were taken to anonymise it.
- Line 226- change “subject” to “participant”
- Line 239- “high” should reader “higher”
- Line 351- reads “affecting” – it should read “effect”
- Line 405 concretizes the literature that adolescents’ intentions to drink are influenced by peers. The authors should state this and justify with a citation.
- Line 425 a word is missing after “drinking”, perhaps “campaigns” so that it reads “stopping drinking campaigns”
