# Supplementary material for: Analysis of related factors for adolescents’ intention to use alcohol in Korea
Source: PLoS One. 2022 Oct 11;17(10):e0275957. doi: 10.1371/journal.pone.0275957 (PMC9553045; doi:10.1371/journal.pone.0275957)
Supplement: Attachment — Submitted filename: Response to Reviewers_round2_reviewer2.docx [file pone.0275957.s008.docx]

| **Editor comment** | **Answer** |
| --- | --- |
|  | It was described based on the lines in this file (Plos one_Revised manuscription_round2_track check file Line) and Changes are marked in blue. |
| Change “liquor commercial” to either “liquor commercials” or “exposure to liquor commercials” | We agree with this suggestion. So based upon the suggestion, we changed the word “liquor commercial”. (Line 42-43, 245, 251, 258, 308, 314, 340, 342, 343) |
| 1. In the abstract, figures for the variable 'grade' are omitted. | We agree with this suggestion. So based upon the suggestion, we added figures for the variable 'grade'. (Line 11-12, 17-18) |
| 2. It is unclear what are protective and risk factors in the way that the results are written up in the abstract. Perhaps group them as such. | We think that all factors could be risk factors. |
| 2. Please decide on the number of decimal points (2 or 3) for the Cronbach's alphas and be consistent. | We decided on the number of decimal points 2 for the Cronbach's alphas and be consistent. (Line 107, 114-115) |
| 2. Figure 1 is shown twice | We removed one Figure 1. (Line 143-144) |
| 3. Check consistency of the variable: "desire to drink alcohol after watching liquor advertisement" throughout the manuscript, sometime 'desire to' is left out. | We added “desire to” throughout the manuscript. (Line 317) |
