# Supplementary material for: Analysis of related factors for adolescents’ intention to use alcohol in Korea
Source: PLoS One. 2022 Oct 11;17(10):e0275957. doi: 10.1371/journal.pone.0275957 (PMC9553045; doi:10.1371/journal.pone.0275957)
Supplement: Attachment — Submitted filename: Review 3-Plos one.docx [file pone.0275957.s009.docx]

**Review 3:**

**Comments**

- Line 44: Change commercial to “commercials and promotes to promote.
- Line 61 change intended to “sought”
- Line 66 change Participant to “Participants” in the heading.
- Line 71 change participant to “participants”
- Line 84 add “a” between the words “were total” and of after the word “total”
- Line 115, check the Cronbach alpha score .9~~43,~~ should read .94
- Line 129 change participant to “participants”
- Line 151 change participant to “participants”
- Line 158 change participant to “participants”
- Line 200 change Characteristics of participant to “ Characteristics of participants”
- Line 246 change liquor commercial to “liquor commercials”
- Line 252 change liquor commercial to “liquor commercials”
- Line 267 does this mean to convey the ease with which purchase alcohol? If so, then change to "ease of alcohol purchase"
- Line 277 change Internet to “internet”
- Line 279 change karaoke to “karaokes”
- Just for Lines 304 and 305, please spell out the acronyms SUA PFD again, to remind the reader of what it means.
- Line 309 change liquor commercial to “liquor commercials”
- Line 315 change liquor commercial to “liquor commercials”
- Line 341 change liquor commercial to “liquor commercials”
- Line 343 add in the words “exposed to” before “liquor commercials”
- Line 344 change liquor commercial to “liquor commercials”
- Line 364 change compared to drink to “compared to drinking”
- Line 365 either change In adolescents to “among adolescents” or change to “in adolescence”
