# Supplementary material for: Analysis of related factors for adolescents’ intention to use alcohol in Korea
Source: PLoS One. 2022 Oct 11;17(10):e0275957. doi: 10.1371/journal.pone.0275957 (PMC9553045; doi:10.1371/journal.pone.0275957)
Supplement: Attachment — Submitted filename: Response to Reviewers_round 3.docx [file pone.0275957.s010.docx]

**Review 3:**

We agree with this suggestion. So based upon the suggestion, We revised and highlight to red.

| **Comment** | **Answer** |
| --- | --- |
| Line 44: Change commercial to “commercials and promotes to promote. | Line 43 : Liquor commercial promotes adolescents’ -> Liquor commercials and promotes to promote adolescents’ |
| Line 61 change intended to “sought” | Line 61 : we intended to provide -> we intended to sought |
| Line 66 change Participant to “Participants” in the heading. | Line 65 : 2.1. Participant -> 2.1. Participants |
| Line 71 change participant to “participants | Line 70 : The participant for this survey -> The participants for this survey |
| Line 84 add “a” between the words “were total” and of after the word “total” | Line 83 : the tool were total 44 items –> the tool were a total of 44 items |
| Line 115, check the Cronbach alpha score .9~~43,~~ should read .94 | Line 114 : Cronbach’s α in this study was .96 (father) and .94 (mother). -> Cronbach’s α in this study was .94 |
| Line 129 change participant to “participants” | Line 128 : participant attend. -> participants attend. |
| Line 151 change participant to “participants” | Line 146 : the research participant was as follows -> the research participants was as follows |
| Line 158 change participant to “participants” | Line 152 : Considering that the participant of the survey -> Considering that the participants of the survey |
| Line 200 change Characteristics of participant to “ Characteristics of participants” | Line 193 : 3.1. Characteristics of participant -> 3.1. Characteristics of participants |
| Line 246 change liquor commercial to “liquor commercials” | Line 239 : being exposed to liquor commercial -> being exposed to liquor commercials |
| Line 252 change liquor commercial to “liquor commercials” | Line 245 : watching liquor commercial -> watching liquor commercials |
| Line 267 does this mean to convey the ease with which purchase alcohol? If so, then change to "ease of alcohol purchase" | Line 259 : as "easy of alcohol purchase" -> as "ease of alcohol purchase" |
| Line 277 change Internet to “internet” | Line 269 : Internet game -> internet game |
| Line 279 change karaoke to “karaokes” | Line 271 : Karaoke -> karaokes |
| Just for Lines 304 and 305, please spell out the acronyms SUA PFD again, to remind the reader of what it means. | Line 296 and 297 : This study identified SUA in drinking and PFD in non-drinking adolescents -> This study identified the sustainable use of alcohol (SUA) in drinking adolescents and the possibility of future drinking (PFD) in non-drinking adolescents |
| Line 309 change liquor commercial to “liquor commercials” | Line 301 : after watching liquor commercial -> after watching liquor commercials |
| Line 315 change liquor commercial to “liquor commercials” | Line 307 : after watching liquor commercial -> after watching liquor commercials |
| Line 341 change liquor commercial to “liquor commercials” | Line 333 : Liquor commercial -> Liquor commercials |
| Line 343 add in the words “exposed to” before “liquor commercials” | Line 335 : being liquor commercial -> being exposed to liquor commercials |
| Line 344 change liquor commercial to “liquor commercials” | Line 336 : after watching liquor commercial -> after watching liquor commercials |
| Line 364 change compared to drink to “compared to drinking” | Line 356 : compared to drink -> compared to drinking |
| Line 365 either change In adolescents to “among adolescents” or change to “in adolescence” | Line 357 : In adolescents -> In adolescence, |
